# Association of Pre-Diagnosis Breast Assessment Practices and Risk Factors with Breast Cancer Stage at Diagnosis: A Single-Center Cross-Sectional Study

**DOI:** 10.3390/jcm15166288

**Published:** 2026-08-14

**Authors:** Halime Seda Küçükerdem, Cengiz Yılmaz, Özden Gökdemir

**Affiliations:** 1 Department of Family Medicine, İzmir City Hospital, İzmir 35540, Türkiye; sedaboz@gmail.com; 2 Department of Medical Oncology, İzmir City Hospital, İzmir 35540, Türkiye; drcengizyilmaz@gmail.com; 3 Department of Family Medicine, Faculty of Medicine, İzmir University of Economics, Sakarya Cd. No. 156, İzmir 35330, Türkiye

**Keywords:** breast cancer, stage at diagnosis, mammography, screening behavior, cross-sectional study

## Abstract

**Objective:** To describe breast assessment before diagnosis among women with breast cancer (BC) and examine its association with stage at diagnosis. **Methods:** This single-center cross-sectional study included 191 women with BC attending a medical oncology clinic. Participants completed a face-to-face questionnaire on breast self-examination (BSE), clinical breast examination (CBE), mammography, and patient characteristics. The stage was obtained from medical records. Mammography history was self-reported, and screening mammography could not be distinguished from diagnostic imaging. Exploratory logistic regression compared stage IV with stages I–III. **Results:** At diagnosis, 41 women (21.5%) had stage IV disease. Regular monthly BSE, regular CBE, and biennial mammography were reported by 8.4%, 3.7%, and 21.4%, respectively. Metastatic disease was present in 7.3% of women who reported biennial mammography and in 25.3% of other women (*p* = 0.013). In the exploratory multivariable model, biennial mammography was associated with lower odds of metastatic disease (adjusted OR 0.242, 95% CI 0.067–0.869), whereas later menarche was associated with higher odds (adjusted OR 1.273, 95% CI 1.017–1.592). Smoking and hormonal medication use also showed inverse associations, but these findings may reflect confounding or selection and should not be viewed as protective. **Conclusions:** Self-reported biennial mammography was associated with earlier stage and lower odds of metastatic disease at diagnosis. This cross-sectional study of women with BC cannot establish causality or screening-program effectiveness.

## 1. Introduction

Breast cancer (BC) is the most commonly diagnosed cancer in women and a leading cause of cancer death worldwide [1,2]. It also accounts for a substantial part of the cancer burden among women in Türkiye [3].

Risk factors for BC include age, family history, genetic susceptibility, reproductive and hormonal exposures, and lifestyle factors [4,5,6]. Stage at diagnosis is a major prognostic factor, and stage IV disease has different treatment goals and outcomes from stages I–III [7].

Mammography is the main screening test for asymptomatic women and is associated with lower BC mortality [8,9]. Routine BSE has not shown a mortality benefit. CBE may detect clinically apparent cancers but does not replace mammography [10,11].

The Turkish national program recommends mammography every 2 years for women aged 40–69 years, together with clinical assessment and counseling [12,13]. Participation in regular mammography remains low in several Turkish studies [14,15].

Reported barriers to screening include limited awareness, concerns about acceptability and access, and lack of a recommendation from a healthcare professional [16]. Few studies have examined breast assessment before diagnosis among Turkish women with BC or its association with stage at diagnosis.

We aimed to describe self-reported BSE, CBE, and mammography before BC diagnosis and examine their associations with stage at diagnosis. The study included only women with BC and therefore did not assess screening sensitivity, mortality, or population-level program effectiveness.

## 2. Methods

### 2.1. Design and Patient Selection

**Figure 1:**
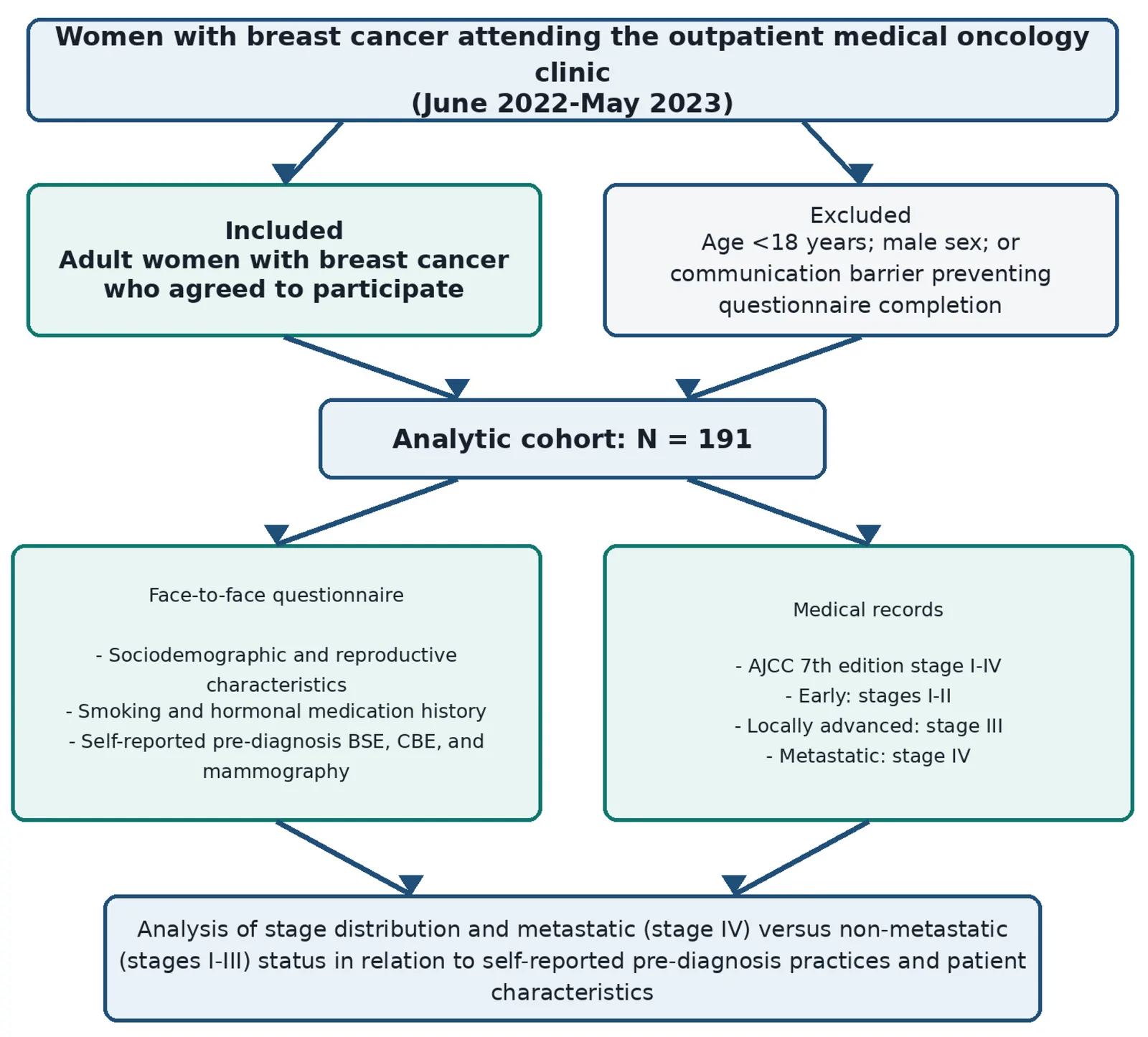
Study design, data sources, and analytic framework.

This single-center cross-sectional study included women aged 18 years or older with BC who attended the outpatient Medical Oncology Clinics at Health Sciences University İzmir Bozyaka Training and Research Hospital between June 2022 and May 2023 and who agreed to participate. A 3 × 2 chi-square test of independence was planned to evaluate the relationship between women’s breast cancer screening status (never screened, irregularly screened, regularly screened) and cancer stage (early stage, advanced stage). In the power analysis carried out using G*Power 3.1.9.7 (Heinrich Heine University Düsseldorf, Düsseldorf, Germany) under parameters of a moderate effect size (w = 0.30) according to Cohen’s standards, a 5% significance level (α = 0.05), and 95% test power (1 − β = 0.95), the minimum required sample size was calculated to be 172. Taking into account possible data losses, dropouts, or incomplete forms that may occur during the prospective study, the sample size was increased by approximately 10%, and the target number of patients was set at 190. Men and patients who were unable to complete the questionnaire due to a communication barrier were excluded (Figure 1). There was no cancer-free comparison group.

### 2.2. Data Collection

Participants completed a face-to-face questionnaire developed after a literature review. It covered demographic characteristics, smoking, hormonal medication use, reproductive history, family history of BC, and BSE, CBE, and mammography before diagnosis. Regular BSE was defined as monthly examination during the previous 5 years. Regular CBE was defined as examination by a physician every 1–3 years before age 40 and annually thereafter. Regular mammography was defined as mammography every 2 years. “Any mammography” included occasional, irregular, and regular mammography. “Regular biennial mammography” was compared with all other patterns.

Mammography history was self-reported and was not checked against imaging records. Exact dates and indications were not recorded, so screening mammography could not be distinguished reliably from diagnostic imaging after symptoms or a palpable finding. Cancer stage was obtained from medical records and classified using the seventh edition of the American Joint Committee on Cancer TNM system [17,18]. Stages I–II were classified as early, stage III as locally advanced, and stage IV as metastatic. BMI was calculated from self-reported weight and height. Five ages at first pregnancy below 14 years were treated as missing. One missing CBE response was excluded from CBE analyses.

### 2.3. Data Analysis

Analyses were performed in SPSS Statistics version 28 (IBM Corp., Armonk, NY, USA). Distributional assumptions were assessed using the Kolmogorov–Smirnov and Shapiro–Wilk tests. Categorical variables were compared with the chi-square test or Fisher’s exact test. Continuous variables were reported as mean ± standard deviation or median (range) and compared with the independent-samples *t* test or Mann–Whitney U test, as appropriate. The logistic regression outcome was stage IV versus stages I–III. The archived SPSS syntax showed that the forward likelihood ratio model considered seven researcher-selected variables: age at menarche, biennial mammography, smoking, hormonal medication use, any family history of BC, maternal BC, and BC in a maternal aunt (entry *p* = 0.05; removal *p* = 0.10). Age at diagnosis and CBE were not included in this model.

Because this was a cross-sectional analysis, the models estimated associations with metastatic presentation among women with BC; they did not estimate the risk of developing BC or causal effects of screening or other exposures. We assessed multicollinearity with variance inflation factors and examined correlations between the dichotomous breast-assessment variables, smoking, and hormonal medication use with phi coefficients. Model performance was summarized using the likelihood ratio test, Nagelkerke R^2^, a 10-group Hosmer–Lemeshow test, area under the receiver operating characteristic curve (AUC), and Brier score. A sensitivity model added age at diagnosis to the final four-variable model. Because only 41 women had stage IV disease and stepwise selection was used, all regression analyses were considered exploratory. We report odds ratios (ORs), 95% confidence intervals (CIs), and two-sided *p* values; *p* < 0.05 was considered statistically significant.

## 3. Results

### 3.1. Clinical and Socio-Demographic Characteristics

The study included 191 women. Mean age at diagnosis was 51.3 ± 11.8 years (range 17–84). At diagnosis, 102 (53.4%) had stage I–II disease, 48 (25.1%) had stage III disease, and 41 (21.5%) had stage IV disease. A total of 132 (69.1%) had less than a high-school education, and 136 (71.2%) were married. A total of 42 (32.5%) reported a family history of BC, 89 (46.6%) reported current or former smoking, and 69 (36.1%) reported previous hormonal medication use. Table 1 presents participant characteristics by metastatic status.

### 3.2. Self-Reported Pre-Diagnosis Breast Assessment Practices

Before diagnosis, 84 (44.0%) participants reported never having mammography, 66 (34.6%) reported occasional or irregular mammography, and 41 (21.4%) reported regular mammography every 2 years (Table 2).

Any BSE was reported by 118 (61.8%) participants, but only 16 (8.4%) reported regular monthly BSE. Any CBE was reported by 82 of 190 participants with a recorded response (43.2%), whereas 7 (3.7%) reported CBE at the frequency defined as regular in this study (Table 2).

Participants who did not report regular biennial mammography could select more than one reason. The most common were having no symptoms (*n* = 76, 37%), having no family history of BC (*n* = 49, 24%), and limited knowledge of the screening program (*n* = 38, 18%). Other reasons were fear of receiving bad news (*n* = 28, 14%), pain (*n* = 11, 5%), and radiation exposure (*n* = 4, 2%).

Neither any BSE nor regular monthly BSE was associated with metastatic status (*p* = 0.630 and *p* = 1.000, respectively). Among participants with a recorded CBE response, metastatic disease was observed in 12 of 82 women who reported any CBE (14.6%) and in 29 of 108 women who reported no CBE (26.9%; *p* = 0.043). CBE was not included in the original archived forward-model candidate set.

Any pre-diagnosis mammography (occasional or regular) was not significantly associated with the AJCC stage, clinical stage, or metastatic status (*p* = 0.214, *p* = 0.184, and *p* = 0.078, respectively; Table 3). In contrast, self-reported regular biennial mammography was associated with the AJCC stage (*p* = 0.006), clinical stage (*p* = 0.004), and metastatic status (*p* = 0.013; Table 3). Metastatic disease was present in 3 of 41 women who reported regular biennial mammography (7.3%) and in 38 of 150 women who reported other mammography patterns (25.3%).

### 3.3. Factors Associated with Metastatic Status

In the univariate analyses, metastatic status was associated with age at menarche (OR 1.286, 95% CI 1.044–1.586), smoking (OR 0.392, 95% CI 0.186–0.826), hormonal medication use (OR 0.354, 95% CI 0.153–0.818), any CBE (OR 0.467, 95% CI 0.222–0.984), and biennial mammography (OR 0.233, 95% CI 0.068–0.797). The forward model retained age at menarche (adjusted OR 1.273, 95% CI 1.017–1.592), smoking (adjusted OR 0.434, 95% CI 0.198–0.955), hormonal medication use (adjusted OR 0.330, 95% CI 0.138–0.788), and biennial mammography (adjusted OR 0.242, 95% CI 0.067–0.869). The inverse associations for smoking and hormonal medication use should not be interpreted as protective (Table 4).

The final model included all 191 women and 41 metastatic events. VIFs ranged from 1.02 to 1.50 in the candidate set and from 1.02 to 1.06 in the final model. Phi coefficients between the breast-assessment variables and smoking or hormonal medication use were all ≤0.202. Model results were as follows: likelihood ratio χ^2^(4) = 24.54 (*p* < 0.001), Nagelkerke R^2^ = 0.187, AUC = 0.750, Brier score = 0.146, and Hosmer–Lemeshow χ^2^(8) = 5.99 (*p* = 0.648).

When age at diagnosis was added to the final model, age was not associated with metastatic status (adjusted OR 0.974, 95% CI 0.944–1.005; *p* = 0.102). The estimate for biennial mammography changed little (adjusted OR 0.238, 95% CI 0.066–0.863; *p* = 0.029).

## 4. Discussion

In this single-center study of 191 women with BC, regular BSE, CBE, and biennial mammography before diagnosis were uncommon. Biennial mammography was associated with earlier stage and lower odds of metastatic disease. Because all participants had BC, these results do not measure screening effectiveness in the general population.

Mean age at diagnosis was 51 years, similar to previous Turkish reports [19]. Age was not associated with metastatic status in either the univariate analysis or the sensitivity model.

One-third of participants reported a family history of BC, but family history was not associated with metastatic status [20]. BMI and the reproductive variables were also not associated with metastatic status. These findings concern stage at diagnosis and do not negate the established role of these factors in BC risk or prognosis.

Only 21.4% reported biennial mammography, 8.4% regular monthly BSE, and 3.7% regular CBE. Other Turkish studies have also reported low mammography uptake [14,15]. However, our rates apply only to women who later received a BC diagnosis and are not population screening rates.

The main reasons for not having regular mammography were the absence of symptoms, a lack of family history, and limited knowledge. We interpreted these barriers within the Turkish screening context, where low mammography uptake has been reported [14,15]. Because we did not measure healthcare access or socioeconomic conditions, we avoided detailed comparisons with dissimilar settings.

The association between biennial mammography and earlier stage is consistent with population-based evidence on mammography screening [21,22,23]. However, the adjusted OR of 0.242 does not show that mammography reduced metastatic risk by 75%. Recall error, diagnostic mammography after symptoms, residual confounding, and selection of patients from an oncology clinic may have affected the estimate.

CBE was associated with less metastatic disease in the unadjusted analysis, but it was not included in the original multivariable model. CBE can detect some cancers missed by mammography [24], but our data do not show an independent effect. BSE was not associated with stage or metastatic status, in line with evidence that routine BSE instruction does not reduce BC mortality [10,25].

Obesity and reproductive and hormonal factors are related to BC risk and, in some settings, prognosis [26,27,28,29]. Our study examined metastatic status at diagnosis, not the risk of developing BC. Null results may reflect limited power, measurement error, or the selected study population.

Smoking and hormonal medication use were inversely associated with metastatic status, but these findings should not be interpreted as biological protection. Because the analysis was restricted to women who had already developed BC, conditioning on case status may have introduced index-event (collider) bias and may partly explain these inverse associations. Residual confounding, selection of patients from an oncology clinic, and differences in healthcare contact or imaging may also have contributed. We did not measure healthcare use directly. The low event count and stepwise model further limit stability; these estimates are exploratory.

## 5. Limitations

This study has several limitations. BSE, CBE, and mammography histories were self-reported and may be affected by recall or misclassification. Imaging dates and indications were unavailable, so screening mammography could not be separated reliably from diagnostic imaging. All participants had BC and attended one oncology clinic; therefore, the findings may not generalize to other patients and cannot estimate screening effectiveness or mortality reduction.

We did not collect data on socioeconomic status, healthcare access, urban or rural residence, diagnostic delay, symptomatic versus screen-detected presentation, breast density, molecular subtype, genetic variants, or alcohol use. Only 41 women had metastatic disease. The candidate variables were researcher-selected rather than prespecified, age at diagnosis and CBE were not included, and stepwise selection was used. The model may therefore be overfitted. It was not internally or externally validated, and its estimates require confirmation in larger population-based studies with verified screening records.

## 6. Conclusions

Regular breast assessment before diagnosis was uncommon in this sample. Self-reported biennial mammography was associated with earlier stage and lower odds of metastatic disease, whereas BSE was not associated with metastatic status. The study does not establish causality or screening effectiveness. Larger population-based studies with verified screening records are needed.

## Figures and Tables

**Table 1:** Clinical and sociodemographic characteristics and self-reported pre-diagnosis breast assessment practices, stratified by metastatic status.

Variables		Overall		Non-Metastatic		Metastatic		*p*
		*n* = 191		*n* = 150		*n* = 41		
		*n*	%	*n*	%	*n*	%	
AJCC stage	Stage I (early)	29	15.2	29	19.3	0	0	
	Stage II (early)	73	38.2	73	48.6	0	0	
	Stage III (locally advanced)	48	25.1	48	32.0	0	0	
	Stage IV (metastatic)	41	21.5	0	0	41	100	
Age at diagnosis, years	Mean ± SD (range)	51.3 ± 11.8 (17–84)		51.7 ± 11.6 (17–84)		50.0 ± 12.7 (24–73)		*0.428*
BMI, kg/m^2^	Mean ± SD (range)	28.9 ± 7.0 (18.3–64.5)		29.1 ± 7.2 (18.3–64.5)		28.2 ± 6.2 (18.7–45.2)		*0.442*
Age at menarche, years	Median (range)	13 (10–18)		13 (10–18)		14 (11–18)		*0.016*
Age at menopause, years (*n* = 152)	Median (range)	47 (29–58)		47 (29–58)		50 (29–54)		*0.723*
Age at first pregnancy, years (*n* = 169)	Median (range)	22 (14–42)		22 (14–39)		20 (16–42)		*0.281*
Number of pregnancies	Median (range)	3 (0–10)		3 (0–10)		3 (0–9)		*0.777*
Total breastfeeding duration, months	Median (range)	24 (0–144)		20 (0–144)		24 (0–144)		*0.484*
Educational level	Below high school	132	69.1	102	68.0	30	73.2	*0.525*
	High school or above	59	30.9	48	32.0	11	26.8	
Married at diagnosis	No	55	28.8	43	28.7	12	29.3	*0.940*
	Yes	136	71.2	107	71.3	29	70.7	
Family history of breast cancer	No	129	67.5	101	67.3	28	68.3	*0.907*
	Yes	62	32.5	49	32.7	13	31.7	
Smoking history before diagnosis	No	102	53.4	73	48.7	29	70.7	*0.012*
	Yes	89	46.6	77	51.3	12	29.3	
Hormonal medication use before diagnosis	No	122	63.9	89	59.3	33	80.5	*0.012*
	Yes	69	36.1	61	40.7	8	19.5	
Any BSE	No	73	38.2	56	37.3	17	41.5	*0.630*
	Yes	118	61.8	94	62.7	24	58.5	
Regular monthly BSE	No	175	91.6	137	91.3	38	92.7	*1.000*
	Yes	16	8.4	13	8.7	3	7.3	
Any CBE (*n* = 190)	No	108	56.8	79	53.0	29	70.7	*0.043*
	Yes	82	43.2	70	47.0	12	29.3	
Any pre-diagnosis mammography	No	84	44.0	61	40.7	23	56.1	*0.078*
	Yes	107	56.0	89	59.3	18	43.9	
Regular biennial mammography	No	150	78.6	112	74.7	38	92.7	*0.013*
	Yes	41	21.4	38	25.3	3	7.3	

Data are n (%) unless otherwise indicated. AJCC, American Joint Committee on Cancer; BMI, body mass index; BSE, breast self-examination; CBE, clinical breast examination. Percentages for CBE use the 190 participants with a recorded response. Five implausible recorded ages at first pregnancy (<14 years) were treated as missing. *p* values are from the independent-samples *t* test, Mann–Whitney U test, chi-square test, or Fisher’s exact test, as appropriate.

**Table 2:** Distribution of self-reported pre-diagnosis breast assessment practices.

Practice	Response Category	*n* (%)
Self-breast examination (BSE)	Not aware of BSE; never performed	52 (27.2)
	Aware of BSE; never performed	21 (11.0)
	Performed occasionally or irregularly	102 (53.4)
	Performed regularly every month	16 (8.4)
Clinical breast examination (CBE)	Not aware of CBE; never underwent	62 (32.5)
	Aware of CBE; never underwent	46 (24.1)
	Underwent occasionally or irregularly	75 (39.3)
	Underwent regularly	7 (3.7)
	Missing response	1 (0.5)
Mammography	Not aware of mammography; never underwent	42 (22.0)
	Aware of mammography; never underwent	42 (22.0)
	Underwent occasionally or irregularly	66 (34.6)
	Reported regular mammography every 2 years	41 (21.4)

BSE, breast self-examination; CBE, clinical breast examination. All responses refer to the period before breast cancer diagnosis; one CBE response was missing.

**Table 3:** Stage at diagnosis according to any self-reported pre-diagnosis mammography and self-reported regular biennial mammography.

Group	Stage/Status	Total *N* = 191 *n* (%)	Any Pre-Diagnosis Mammography (Occasional or Regular)			Regular Biennial Mammography (Every 2 Years)		
			No *n* = 84	Yes *n* = 107	*p*	No *n* = 150	Yes *n* = 41	*p*
AJCC stage	Stage I	29 (15.2)	9 (10.7)	20 (18.7)	*0.214*	18 (12.0)	11 (26.8)	*0.006*
	Stage II	73 (38.2)	31 (36.9)	42 (39.3)		53 (35.3)	20 (48.8)	
	Stage III	48 (25.1)	21 (25.0)	27 (25.2)		41 (27.3)	7 (17.1)	
	Stage IV	41 (21.5)	23 (27.4)	18 (16.8)		38 (25.3)	3 (7.3)	
Clinical stage	Early	102 (53.4)	40 (47.6)	62 (57.9)	*0.184*	71 (47.3)	31 (75.6)	*0.004*
	Locally advanced	48 (25.1)	21 (25.0)	27 (25.2)		41 (27.3)	7 (17.1)	
	Metastatic	41 (21.5)	23 (27.4)	18 (16.8)		38 (25.3)	3 (7.3)	
Metastatic status	Non-metastatic	150 (78.5)	61 (72.6)	89 (83.2)	*0.078*	112 (74.7)	38 (92.7)	*0.013*
	Metastatic	41 (21.5)	23 (27.4)	18 (16.8)		38 (25.3)	3 (7.3)	

Data are n (column %). ‘Any mammography’ includes occasional/irregular or regular pre-diagnosis mammography. Regular biennial mammography means every 2 years. Mammography indication (screening vs. diagnostic) was not verified.

**Table 4:** Exploratory logistic-regression analysis of factors associated with metastatic disease at diagnosis.

Metastatic Versus Non-Metastatic	Univariate Model			Multivariate Model		
	*OR*	*95% CI*	*p*	*OR*	*95% CI*	*p*
Age at diagnosis, years	0.988	0.960–1.018	0.426			
BMI	0.979	0.928–1.033	0.440			
Below high-school education	1.283	0.593–2.776	0.526			
Married at diagnosis	0.971	0.454–2.077	0.940			
Family history of breast cancer (F)	0.957	0.456–2.008	0.907			
Mother with breast cancer (F)	0.243	0.031–1.904	0.178			
Maternal aunt with breast cancer (F)	0.648	0.138–3.047	0.583			
Age at menarche, years (F)	1.286	1.044–1.586	0.018	1.273	1.017–1.592	0.035
Number of pregnancies	1.000	0.849–1.179	0.995			
Age at first pregnancy, years	0.972	0.904–1.045	0.445			
Total breastfeeding duration, months	1.008	0.997–1.019	0.137			
Age at menopause, years	1.004	0.941–1.071	0.911			
Smoking history before diagnosis (F)	0.392	0.186–0.826	0.014	0.434	0.198–0.955	0.038
Hormonal medication use before diagnosis (F)	0.354	0.153–0.818	0.015	0.330	0.138–0.788	0.013
Any BSE before diagnosis	0.841	0.416–1.700	0.630			
Regular monthly BSE before diagnosis	0.832	0.225–3.071	0.782			
Any CBE before diagnosis	0.467	0.222–0.984	0.045			
Any mammography before diagnosis	0.536	0.267–1.078	0.080			
Regular biennial mammography before diagnosis (F)	0.233	0.068–0.797	0.020	0.242	0.067–0.869	0.030

Exploratory binary logistic regression; outcome = metastatic (stage IV) versus non-metastatic (stages I–III). (F) indicates a variable entered into the original forward likelihood ratio model. OR, odds ratio; CI, confidence interval. Binary ORs compare yes with no; education compares below high school with high school or above. Univariate models used available cases (age at first pregnancy, *n* = 169; CBE, *n* = 190); final model, *n* = 191 with 41 events.

## Data Availability

All data associated with this paper are available from the corresponding author upon reasonable request.
